# Supplementary material for: Tuning dCas9's ability to block transcription enables robust, noiseless knockdown of bacterial genes
Source: Mol Syst Biol. 2018 Mar 8;14(3):e7899. doi: 10.15252/msb.20177899 (PMC5842579; doi:10.15252/msb.20177899)
Supplement: Supplementary file 1 — Appendix [file MSB-14-e7899-s001.pdf]

Appendix to the manuscript:

Tuning dCas9's ability to block transcription enables robust, noiseless knockdown of bacterial genes

Antoine Vigouroux, Enno Oldewurtel, Lun Cui, David Bikard and Sven van Teeffelen

Table of contents

1. A novel method of allelic exchange using CRISPR/Cas9
2. Autorepressor model
3. Appendix figures
4. Appendix tables
5. Appendix references

1 A novel method of allelic exchange using CRISPR/Cas9

To make scar-less genome modifications easier, we developed a new method which relies on the integration of a non-replicating plasmid through homologous recombination followed by backbone removal promoted by Cas9 cleavage in the antibiotic resistance gene (Appendix Fig S9). This method was inspired from a similar strategy that makes use of the I-SceI nuclease instead of Cas9 (Posfai *et al.*, 1999).

The chromosomal modifications are carried out using a plasmid typically made by assembling together four PCR fragments: 1) a chloramphenicol-resistant backbone with the R6K conditional origin of replication (such as pSW23t), 2) a homology region of about 1 kbp matching the sequence before the desired site of insertion, 3) a reporter sequence, possibly using a linker for protein fusions, or a stop codon and a ribosome binding site for mRNA fusions, 4) a second homology region of 1 kbp matching the sequence after the site of insertion, i.e. the beginning of the gene of interest.

This plasmid is electroporated in the recipient strain. Cells that have integrated the plasmid by homologous recombination (HR) are selected by plating on chloramphenicol. Either one or the other homology arm will be used for HR. The two insertion orders can be told apart by colony PCR with one primer in the backbone and one primer on the chromosome. We then pick a colony from each insertion order, dilute them in 500 ul of LB and grow them during 1h30, after which cells are centrifuged and recovered in 100 ul of TSS (Transformation and Storage Solution) (Chung, Niemela and Miller, 1989).The cells are then transformed with plasmid pAV10, expressing Cas9 under the control of a DAPG-inducible promoter and a sgRNA targeting the *cat* gene of the suicide vector, followed by plating on LB agar with kanamycin and DAPG at 30°C. Cleavage by Cas9 in the chromosome triggers the resolution of the co-integrate in two possible ways: recombination can either restore the wild-type locus, or lead to the desired seamless modification. Several colonies from each insertion order are re-streaked on LB agar without kanamycin and grown at 42°C to eliminate thermo-sensitive pAV10 and obtain clonal populations. Excising the backbone from both insertion orders greatly increases the chances of finding a good clone, as for each order the recombination might be biased towards a particular

outcome. We recommend to re-streak a minimum of 2 colonies for each order. Finally, we screen for edited clones by colony PCR with one primer on each side of the insert.

As a demonstration for this method, we created plasmid pAV25, allowing integration of a PAmCherry-PBP2 fusion in the native locus using a variant of the *PAmCherry* gene that can be targeted by our collection of guide RNAs. Without changing the amino-acid sequence of PAmCherry, the nucleotide sequence of the ORF was silently modified so the *mCherry*-targeted guide RNAs would also be effective on the photo-activatable version. Thus we produce a strain where the concentration of PBP2 and RodA can be controlled like in Figure 6, but whose mCherry can be activated by light for single-molecule tracking.

2 Autorepressor model

In this section we present two simplified models of the PhlF-autorepressor. In both cases, PhlF expression is represented by the straight-forward reaction kinetics

$$\dot{R} = f(R,c)\beta\alpha - \lambda R\,, \tag{2.1}$$

where R is the PhlF concentration (including proteins bound in dimers or other multimers), α is the rate of the protein production in the absence of any feedback or CRISPR knockdown, β is the passage probability due to dCas9, λ is the protein dilution rate, and $f(R,c)$ characterises the feedback strength as a function of protein concentration R and DAPG concentration c. During steady-state conditions PhlF expression is thus determined by the self-consistent equation

$$f(r,c)\beta - r = 0\,. \tag{2.2}$$

Here, $r = R/(\alpha/\lambda)$ is the PhlF concentration normalised with respect to the PhlF concentration in the absence of feedback or CRISPR-knockdown.

2.1 Repression modelled by Hill-function

In the first model (model a), auto-repression through PhlF proteins is represented by a Hill function

$$f(r,c) = 1/\left[1 + (r/k(c))^n\right]\,. \tag{2.3}$$

Here, cooperativity of two or more PhlF proteins in dimers or multimers is subsumed in the Hill exponent n and any DAPG-dependent reduction of repression is modelled by an effective binding constant $k(c)$. Here, we assume that DAPG binding and unbinding to PhlF proteins is much more rapid than the dynamics of PhlF expression and dilution.

Together, Eqs. (2.2, 2.3) lead to a self-consistent equation for r:

$$r\left[1 + (r/k(c))^n\right] - \beta = 0\,. \tag{2.4}$$

In Appendix Figures S11A and S11B this model is fit to the experimental data of GFP expression assuming two different values of $n = 1,2$. The model with $n = 2$ fits the data well for 0 and 5 uM DAPG, but not for 50 uM. On the contrary, the model with $n = 1$ fits the data well for 50 uM, but not for 0 and 5 uM.

2.2 Repression modelled by dimer formation

Since PhlF is known to dimerise *in vitro* (Abbas *et al.*, 2002) and thought to bind the operator site as dimer we hypothesised that the different Hill coefficients observed could come about because PhlF proteins are predominantly found as monomers at low DAPG concentrations, thus rendering dimer concentration proportional to r^2, while they are mostly found as dimers at high DAPG concentration, thus rendering dimer concentration proportional to r. We thus formulated a simple model (model b) of dimer-based repression

$$f(r,c) = 1/\left[1 + d/K(c)\right] , \tag{2.5}$$

where d is the concentration of all PhlF dimers, normalised with respect to the maximum number of PhlF proteins (α/λ), and $K(c)$ is the DAPG-dependent effective binding constant of dimers, considering that only a sub-fraction of dimers can efficiently bind the operator. During steady-state conditions the concentration of dimers is determined by

$$\dot{d} = k_{\mathrm{on}}(r - 2d)^2 - k_{\mathrm{off}}d = 0\,, \tag{2.6}$$

where k_{on} and k_{off} are binding and unbinding constants. Introducing the parameter $a = k_{\mathrm{off}}/k_{\mathrm{on}}$, we can solve Eq. (2.6) for d:

$$d = \frac{1}{8}\left(a + 4r - \sqrt{a^2 + 8ar}\right) . \tag{2.7}$$

For low PhlF concentrations ($r \ll a$), dimer concentration is approximately given by $d \approx r^2/a$, which is equivalent to model a with a Hill coefficient of $n = 2$. For values $r \gg a$, dimer formation is approximately given by $d \approx r/2$, which corresponds to a Hill coefficient of $n = 1$. We thus wondered whether the transition in Hill coefficients observed for different DAPG concentrations could come about by a concomitant change of PhlF concentration alone, i.e., if the dimerisation binding constant a remains independent of DAPG concentration. We thus fit the model Eqs. (2.6, 2.7) to the experimental data, using a single binding constant a for all DAPG concentrations but different values of $K(c)$, which reflect different fractions of dimers being capable of binding the operator site.

Empirically, we found for a value of $a = 0.15$ that the increasing PhlF concentration upon increasing DAPG concentration can partially explain the transition in Hill coefficient (Appendix the S11C), while lower or higher values of a provide better fits for the regimes of low or high DAPG concentrations, respectively, but not for both regimes. However, it appears that the transition is more sudden in the experimental data than in the fit. The discrepancy between model and experimental data can be alleviated by rendering the dimerisation constant a DAPG-dependent, with a higher value of $a \approx 2$ for low DAPG concentrations (0 uM and 5 uM) and a low value of $a \approx 0.01$ for high DAPG concentrations (50 uM) (Appendix Fig S11D).

The molecular mechanism underlying the DAPG-dependent binding constant is not understood yet. One potential scenario (possibly among others) is the following: DAPG could effectively stabilise the fraction of dimers that are capable to repress the promoter. A stabilisation of operator-binding dimers through DAPG would be surprising, as DAPG is thought to inhibit operator binding. However, it is conceivable that 'UB'-dimers, where one of the two protomers is DAPG-bound (B) while the other is unbound (U), still bind to the operator, possibly with reduced affinity or increased unbinding rate. These dimers could be stabilised at high DAPG concentrations due to direct or indirect effects of DAPG binding on dimer formation. One possible explanations is the following: If dimers of both

protomers DAPG-bound ('BB'-dimers) were energetically unfavourable to form, the pool of DAPG-bound monomers would be high at high DAPG levels. This pool would then effectively facilitate dimerisation of 'UB'-dimers. We note that this hypothesis remains highly speculative and other mechanisms might be responsible for the transition in Hill coefficients observed.

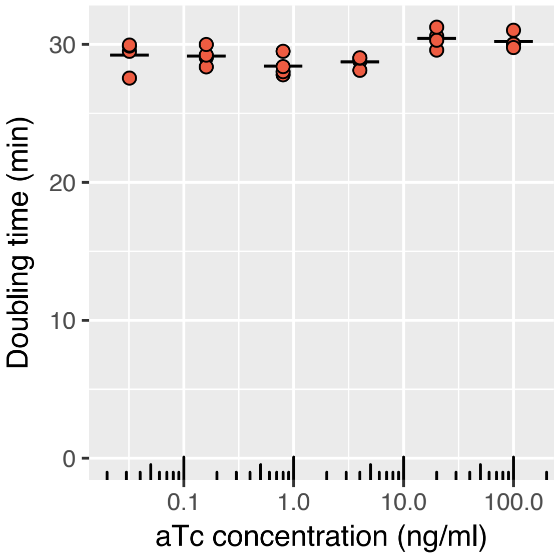

Appendix Figure S1 – Expression of dCas9 from P_{tet} with up to 100 ng/ml of aTc does not produce growth defects. Here the doubling time is measured on the strain AV03 with a non-targeting CRISPR array, in LB at 30°C.

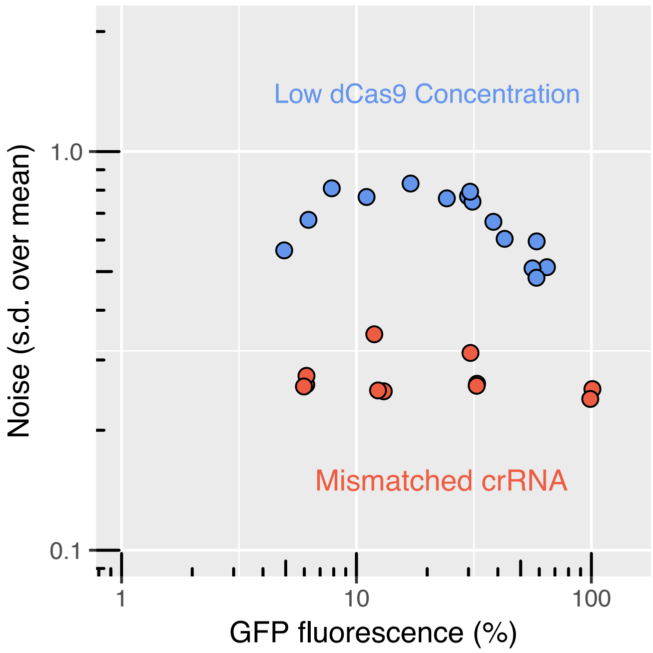

Appendix Figure S2 – GFP is expressed from P_{127} promoter and repressed using dCas9. For a given expression level, the noise is ~3 times lower if expression is tuned by guide RNA complementarity than if expression is tuned by changing dCas9 induction level. The points corresponding to maximal repression (spacer without mismatch at the highest induction level of dCas9) are omitted, as their exact noise could not be quantified accurately using our setup (high-throughput microscopy). Fluorescence values are population means, as a percentage of the fluorescence of P_{127}-*sfGFP* in a strain with no repression. S.d.: standard deviation.

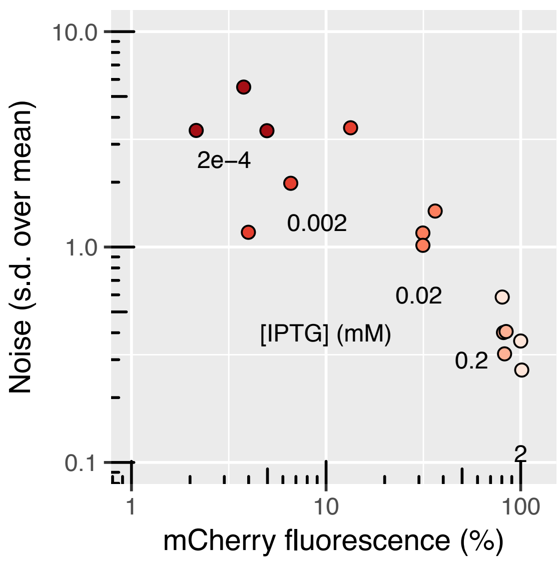

Appendix Figure S3 – Noise in mCherry concentration as a function of expression level from a P_{Lac}-inducible promoter.

mCherry is expressed from P_{Lac} in a Δ*lacY* background and its concentration is changed using different concentrations of IPTG. Fluorescence values are population averages, as a percentage of the fluorescence of P_{Lac}-*mCherry* in a strain with no repression and 2 mM of IPTG. This measurement was done by fluorescence microscopy using agarose pads, as expression from P_{Lac} is much lower than expression from P_{127}.

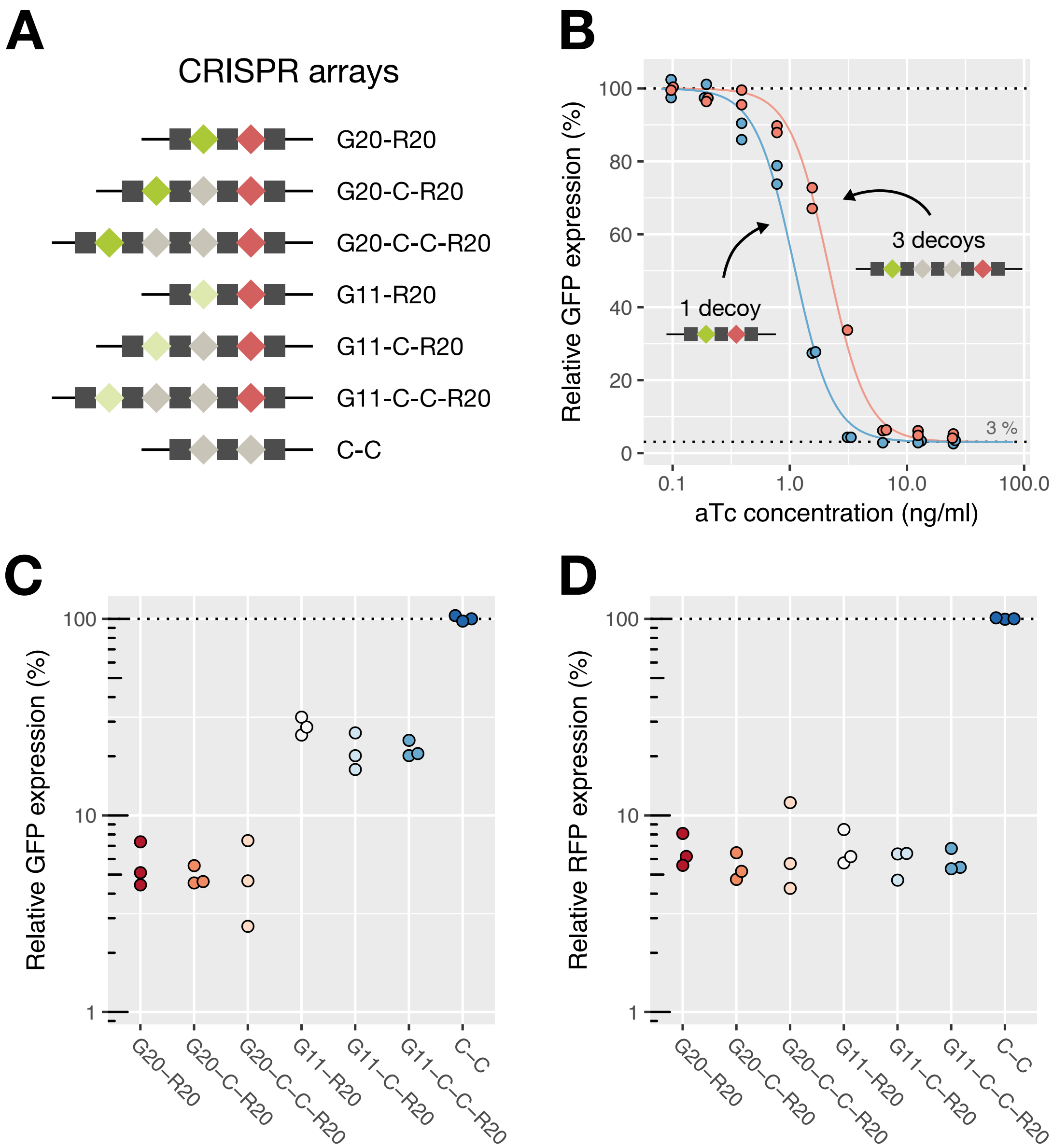

Appendix Figure S4 – At least 4 mismatched guide RNAs can be expressed at the same time while saturating the target.

A: Schematics of the CRISPR arrays used in this experiment. G20 targets the *sfGFP* ORF with full complementarity (20bp), G11 targets it with 11bp of complementarity (9 mismatches), R20 targets the *mCherry* ORF with full complementarity. The C guides have no target in *E. coli*'s chromosome.

B: Flow cytometry measurement of GFP fluorescence when dCas9 is induced with different amounts of aTc. For a low enough aTc concentration, we leave the saturation regime and enter the concentration-dependent regime. Adding other spacers (decoys) to the CRISPR array decreases the concentration of active complex. Therefore, the concentration of aTc required to reach the saturation regime is higher with 3 decoys than with 1 decoy.

C: With 100 ng/ml of aTc, at least 4 different spacers can be added to the array without leaving the saturation regime. This is true even with a mismatched crRNA (G11).

D: The last guide RNA of the CRISPR array (R20) with four spacers is still expressed highly enough to reach saturation of the target and repress mCherry strongly.

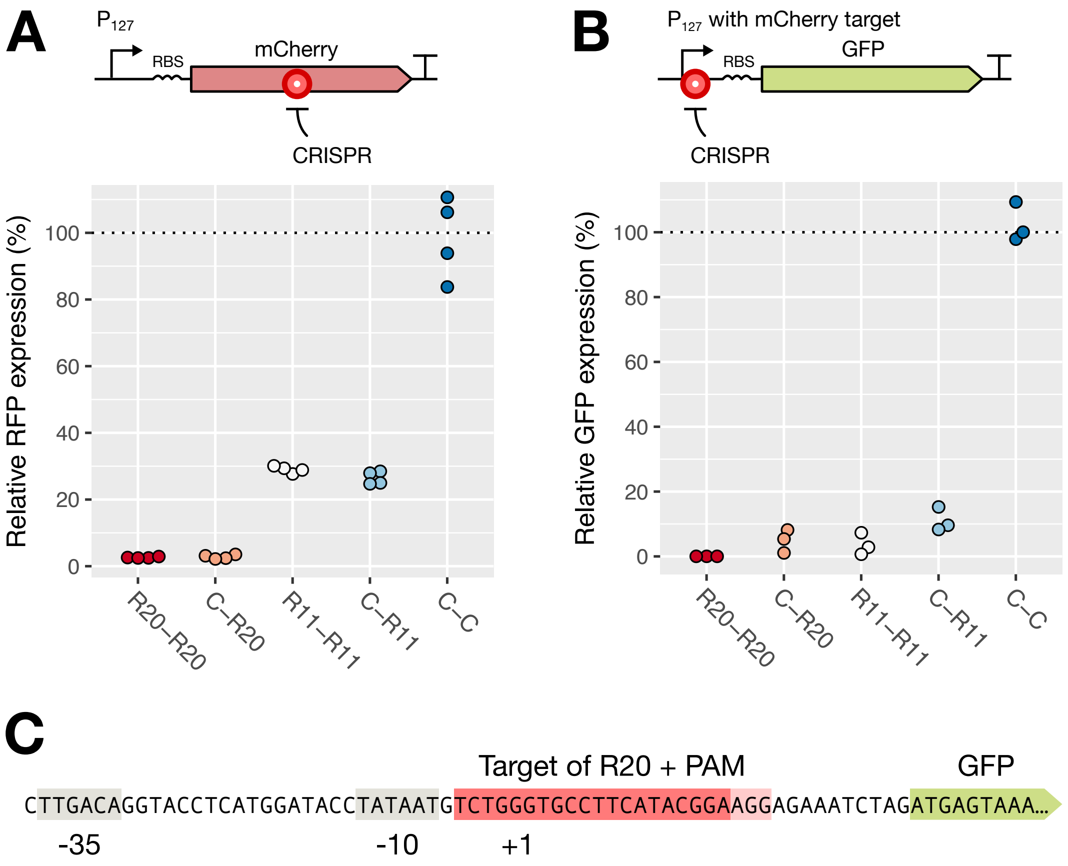

Appendix Figure S5 – Targeting the same sequence inside the ORF or inside the promoter region shows qualitatively different behavior.
Comparison between two strains with the same target either inside the *mCherry* ORF (A; reproduced from Figure 2B) or inside the promoter sequence to *gfp* (B). In the new strain (sequence indicated in C), the P_{127} promoter was modified to include the target sequence (red) otherwise found inside the mCherry ORF. This way, the R20-R20, C-R20, R11-R11 and C-R11 CRISPR arrays used previously (panel a) can bind to the promoter region in this strain, but not to the *gfp* ORF. As dCas9 now blocks transcription initiation rather than elongation, it cannot be kicked out by the RNAP. Accordingly, repression is stronger for low levels of complementarity but dependent on dCas9-complex concentration.

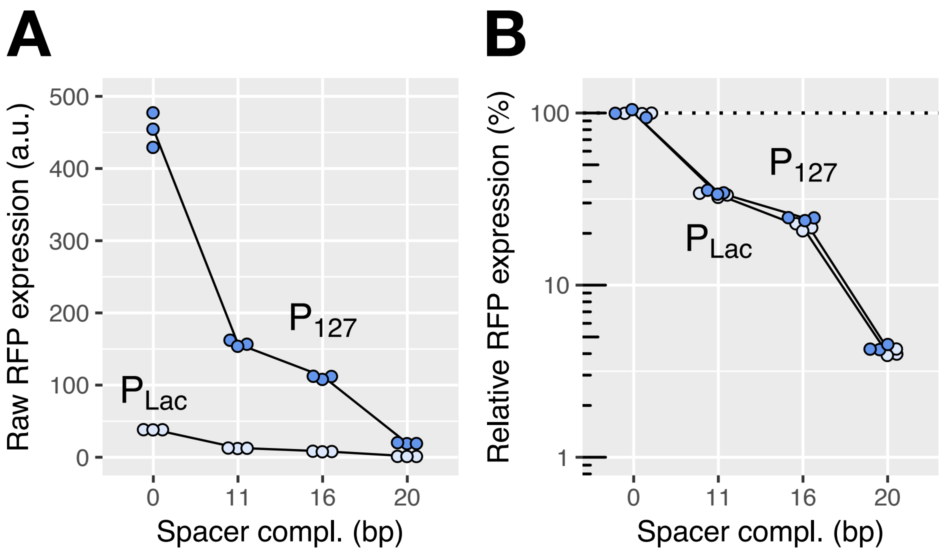

Appendix Figure S6 – Repression by dCas9 is independent of promoter strength over a wide range of transcription rates.

A: Raw RFP expression measured by flow cytometry for P_{127} (also used in Fig 3) and P_{Lac} in the presence of 1 mM of IPTG and repressed using 4 different guide RNAs in saturating conditions. In these conditions, the native expression from P_{Lac} is about 12 times weaker than P_{127}.

B: Relative GFP expression normalized with respect to the non-targeting spacer. The relative expression levels are similar for both promoters. Together with Fig 3, this shows that the independence on transcription rate applies also at a low expression level.

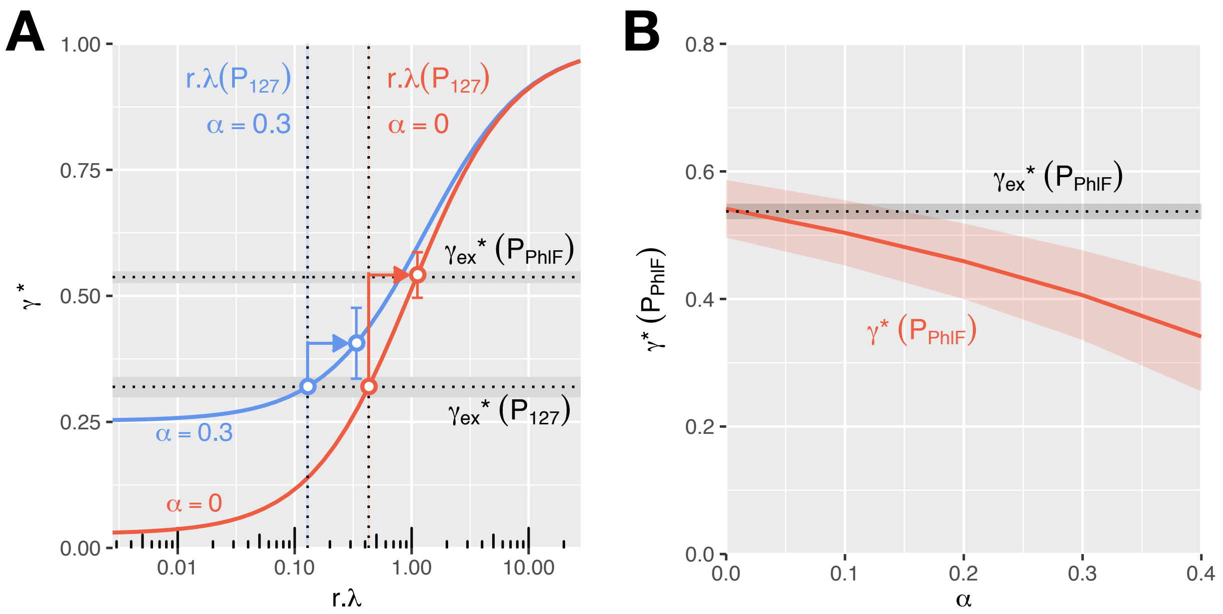

Appendix Figure S7 – Predictions from the kick-out model of CRISPR knockdown: The effect of promoter strength and dCas9 transcription-independent unbinding on gene repression at low and intermediate dCas9 concentrations in the case of full complementarity.

A: The relative GFP expression γ^* is plotted as a function of the kick-out rate $r\lambda$, where λ is the ratio of the rate of transcription-induced dCas9 displacements (given by the product of transcription initiation rate γ_0 and the ejection frequency δ) over the rate of dCas9 rebinding ($[\text{dCas9}]k_{on}$), and r = 1-P(stop) is the probability of dCas9 leaving the target upon collision with the RNAP (see Appendix text for details). The red and blue curves are generated based on the kick-out model and correspond to zero (red) and finite (blue) rates of transcription-independent dCas9 unbinding. Here, α is the transcription-independent unbinding rate normalized by the rebinding rate. The relative expression of the P_{127} promoter $\gamma_{ex}^*(P_{127})$ (the lower of the two horizontal lines, see also Figure 3C) is used as a reference to infer the unknown kick-out rate $r\lambda(P_{127})$ as the intersection point of the two lines (indicated as the lower blue and red open circles). Predicted values for GFP expression from the P_{PhlF}-promoter are indicated by open circles with error bars corresponding to 95% confidence intervals. The experimental GFP expression from the P_{PhlF}-promoter $\gamma_{ex}^*(P_{PhlF})$ (the higher of the two horizontal lines) is predicted correctly by the model if the transcription-independent unbinding rate is assumed to be $\alpha = 0$, but not if $\alpha = 0.3$.

B: Predicted and measured relative expression of P_{PhlF}-*gfp* as a function of α. Only the values of α lower than 0.14 show agreement between prediction and measurement, indicating that transcription-independent unbinding of dCas9 occurs at a rate much smaller than the rate of dCas9 rebinding and smaller than the rate of dCas9 being kicked out by the RNAP.

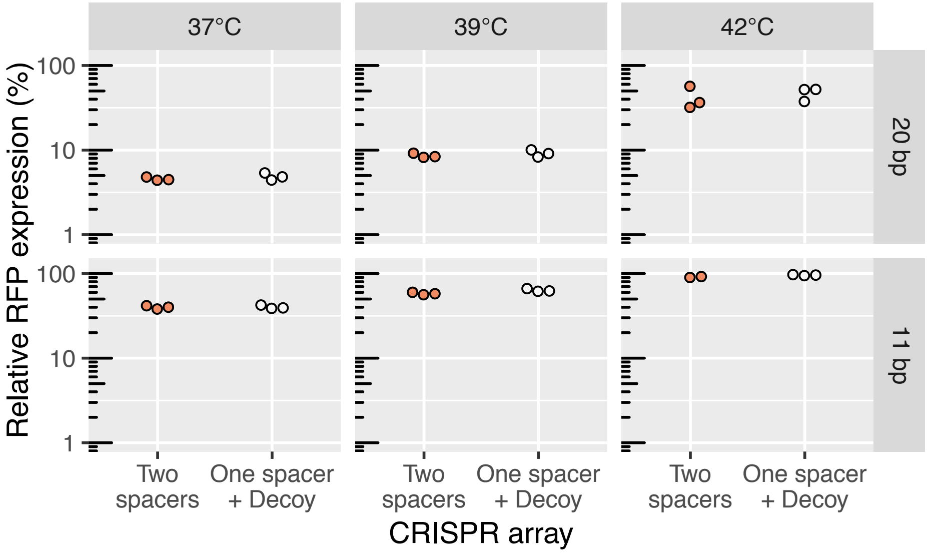

Appendix Figure S8 – Relative RFP expression for different temperatures with either 20 or 11 bp of complementarity, in the presence or absence of a decoy guide RNA.

RFP expression levels are first normalized with respect to a constitutively expressed GFP reporter. RFP/GFP ratios are then normalized with respect to the non-targeting CRISPR array. Doubling of dCas9 concentration does not affect the repression strength at any temperature even with partial complementarity.

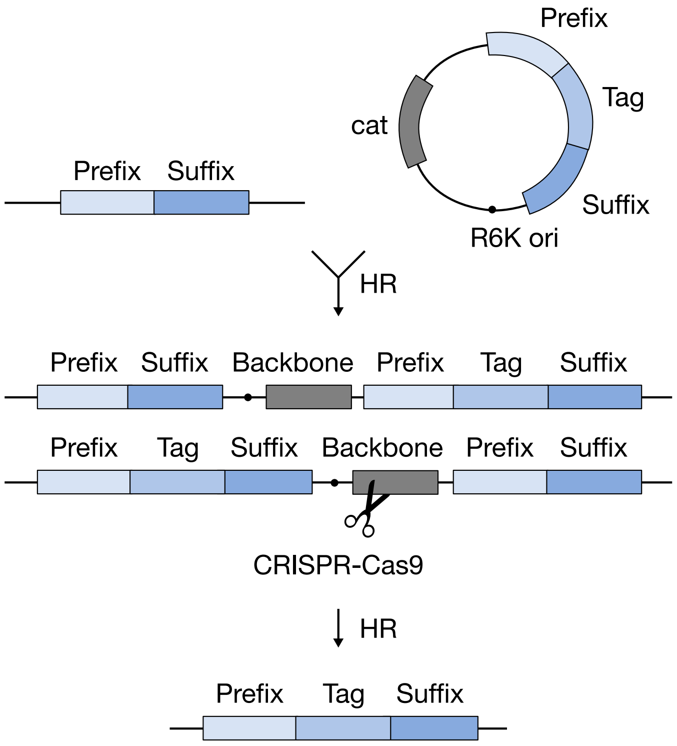

Appendix Figure S9 – Overview of the allelic exchange procedure to integrate a reporter in front of a target gene, using CRISPR/Cas9-triggered excision.
HR: Homologous recombination. Ori: Origin of replication. *cat*: chloramphenicol acetyl-transferase. This procedure is described in details in Appendix text, section 1.

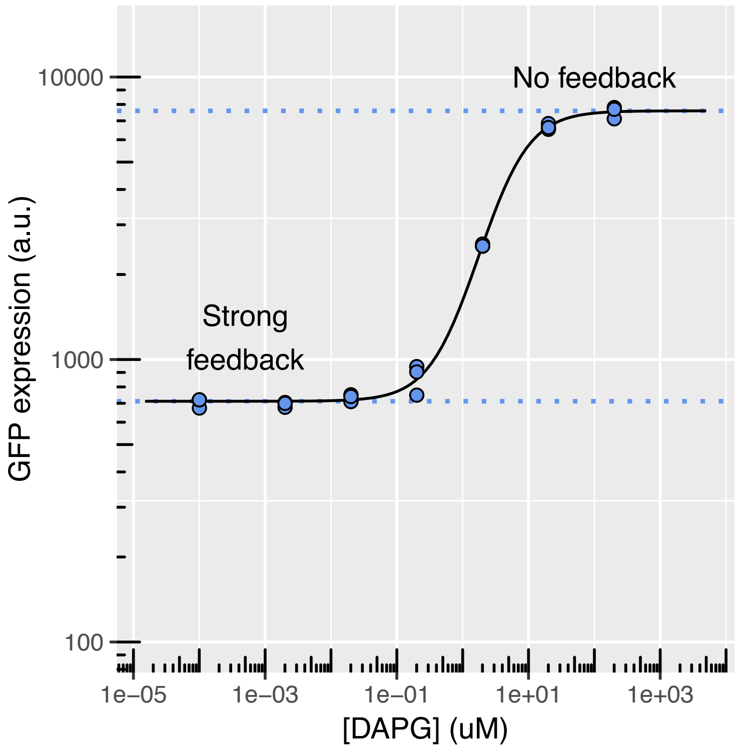

Appendix Figure S10 – Steady-state GFP expression levels with GFP expressed from the feedback loop as a function of the concentration of DAPG, modulating the strength of the feedback. Each point represents one biological replicate. The black line is a fit of the data using a Hill function. The Hill coefficient was calculated to be 1.25±0.07.

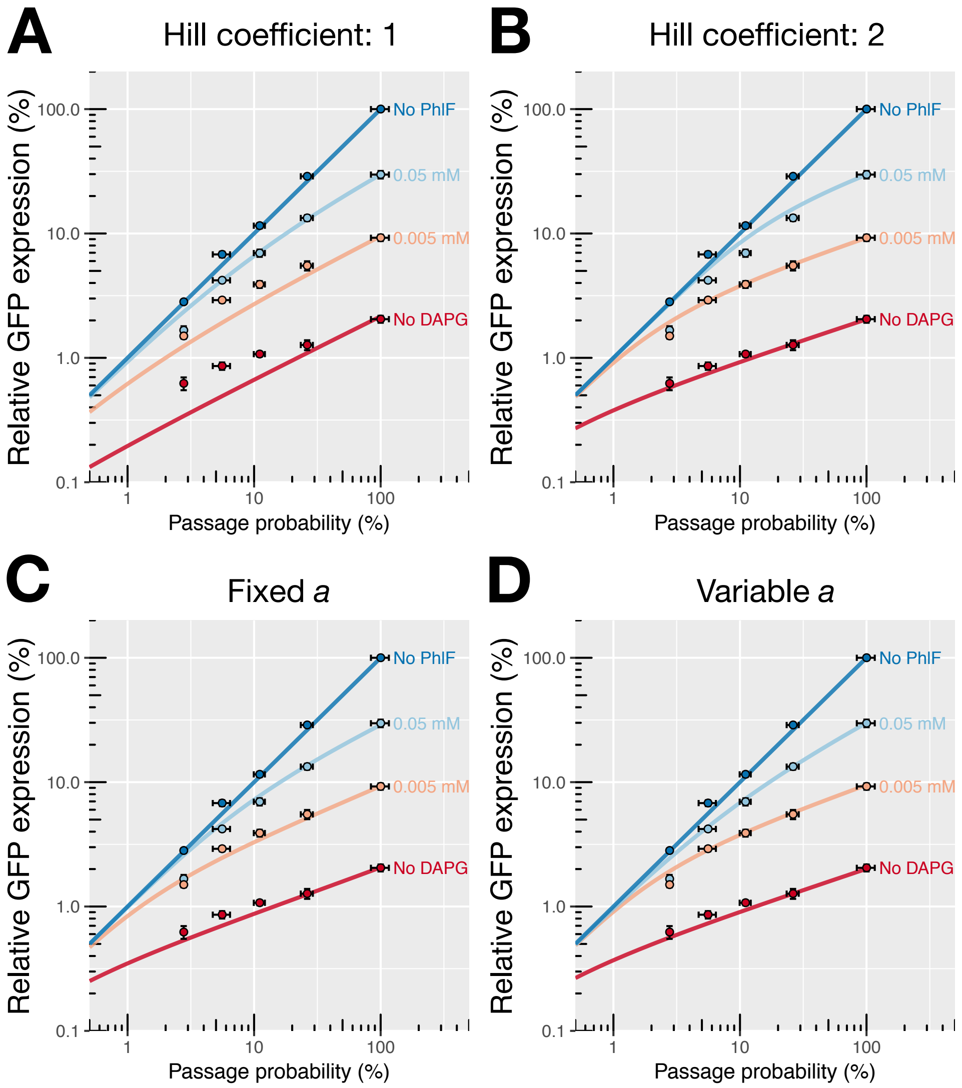

Appendix Figure S11 - Different mathematical models for the PhlF auto-repressor. Points represent relative GFP expression levels as shown in Figure 4. Solid lines represent the values predicted by the different models. a: The transcriptional response of P_{PhlF} is modeled as a Hill function with Hill coefficient n = 1 (A) or n = 2 (B) (model a). b: Biophysical model accounting for PhlF binding to DAPG and its dimerization with either fixed (C) or variable (D) dimerization binding constant a (model B). See Appendix text for more details.

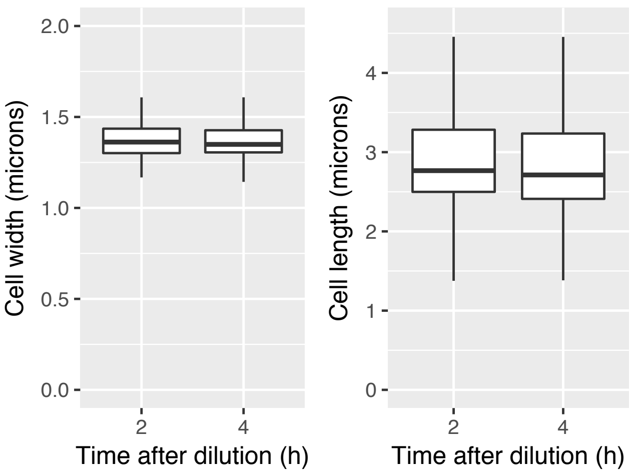

Appendix Figure S12 – **Upon partial depletion of PBP2 and RodA by CRISPR knockdown, the cells maintain their altered morphology stably over time.** The *mCherry-mrdA* fusion gene was repressed using a guide RNA with 18 bp of complementarity (lowering *mrdAB* to 20% of the native level) then the culture was maintained in exponential phase. At two time points (after 2h and after 4h) a sample of the culture was fixed and imaged by microscopy.

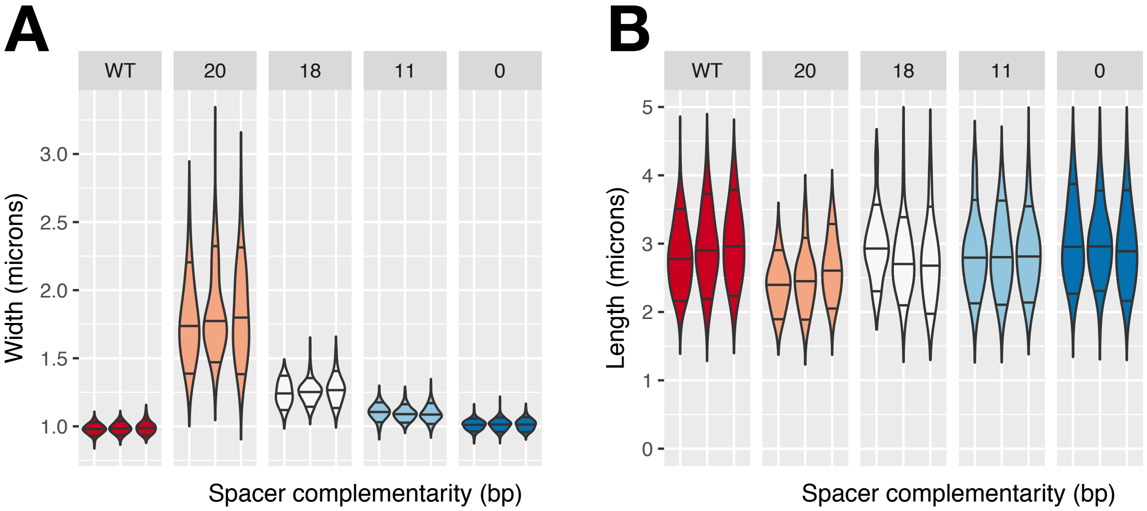

Appendix Figure S13. Distribution of cell diameter (A) and cell length (B) for different levels of *mrdAB* repression. Horizontal lines indicate, from top to bottom, the 90th percentile, the median and the 10th percentile. WT: wild-type.

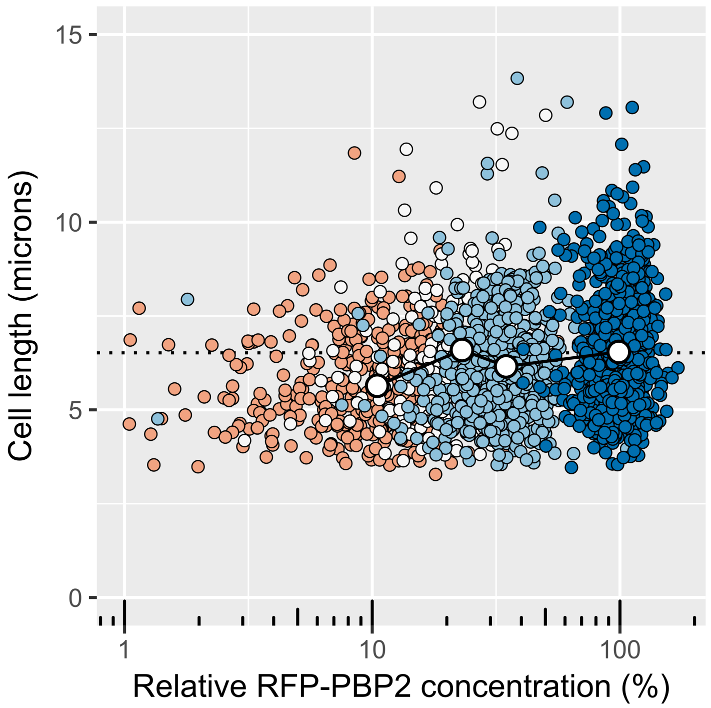

Appendix Figure S14. Cell length as a function of the cellular concentration of RFP-PBP2. Each color represents the population obtained with one CRISPR plasmid. The connected white dots represent the population averages (mean of 3 biological replicates). The dotted line represents the mean cell length for wild-type *E. coli* (mean of 3 replicates).

Table S1: New strains used in this study and the successive plasmid transformations used to make them from their parental strain. All strains derive from MG1655 *E. coli*, except AV08 that derives from TKL130 (Lee *et al*, 2014).

Name	Genotype	Construction
AV01	186::P_{tet}-*dcas9*	pAV03 → pE-FLP
AV02	186::P_{tet}-*dcas9*,<br>HK022::P_{127}-*sfgfp*	pAV03 → pE-FLP → pAV01 → pE-FLP
AV03	186::P_{tet}-*dcas9*,<br>HK022::P_{127}-*sfgfp*,<br>λ::P_{127}-*mcherry*	pAV03 → pE-FLP → pAV01 → pE-FLP → pAV02 → pE-FLP
AV04	186::P_{tet}-*dcas9*,<br>λ::P_{127}-*mcherry*	pAV03 → pE-FLP → pAV02 → pE-FLP
AV06	186::P_{tet}-*dcas9*,<br>HK022::P_{127}-*sfgfp*,<br>λ::P_{lac}-*mcherry*	pAV03 → pE-FLP → pAV01 → pE-FLP → pAV04 → pE-FLP
AV08	*mrdA*::*mcherry-mrdA*,<br>λ::P_{tet}-*dcas9*	pCas → pAV07 → pLC97 → pE-FLP
AV14	186::P_{tet}-*dcas9,*<br>λ::P_{phlf}-*sfgfp-phlF*	pAV03 → pE-FLP → pAV14 → pE-FLP
AV27	186::P_{tet}-*dcas9,*<br>*mrdA*::*pamcherry-mrdA*<br>(repressible variant)	pAV03 → pE-FLP → pAV25 → pAV10
AV75	186::P_{tet}-*dcas9*,<br>HK022::P_{127}-*sfgfp*,<br>λ ::P_{lac}-*mcherry*, Δ *lacY*	pAV03 → pE-FLP → pAV01 → pE-FLP → pAV04 → pE-FLP → P1 from JW0334 (Baba *et al*, 2006) → pE-FLP
AV76	186::P_{tet}-*dcas9*,<br>HK022::P_{127}(R20 target)-<br>*sfgfp*	pAV03 → pE-FLP → pAV81 → pE-FLP

Table S2: Plasmids constructed or used for this study and the corresponding assembly fragments (either PCR products or digested plasmids). In brackets: Addgene IDs.

Name	Description	PCR primers or restriction enzyme		Template	Reference
pAV01	P_{127}-*sfgfp* integration in HK022 site	V1	V2	pDB127	(Bikard *et al*, 2013)
		V3	V4	pHC942	(Cho *et al*, 2016)
		EcoR1	Pst1	pIT5-KH (45983)	(St-Pierre *et al*, 2013)
pAV02	P_{127}-*mcherry* integration in λ site	V1	V40	pDB127	(Bikard *et al*, 2013)
		V38	V39	pFB262	(Bendezú *et al*, 2009)
		EcoR1	Pst1	pIT5-KL (45984)	(St-Pierre *et al*, 2013)
pAV03	P_{tet}-*dcas9* integration in 186 site	LC100	LC283	pDB275	(Depardieu *et al*, 2016)
		LC284	LC285	pdCas9-bacteria	(Qi *et al*, 2013)
		EcoR1	Pst1	pIT5-KO (45985)	(St-Pierre *et al*, 2013)
pLC97*	P_{tet}-*dcas9* integration in λ site	LC100	LC283	pDB275	(Depardieu *et al*, 2016)
		LC284	LC285	pdCas9-bacteria	(Qi *et al*, 2013)
		EcoR1	Pst1	pIT5-KL (45984)	(St-Pierre *et al*, 2013)
pAV04	P_{lac}-*mcherry* integration in λ site	V75	V76	MG1655	
		V77	V78	pFB262	(Bendezú *et al*, 2009)
		EcoR1	Pst1	pIT5-KL (45984)	(St-Pierre *et al*, 2013)
pAV06	Replaces *pamcherry-mrdA* with *mcherry-mrdA*	V67	V68	TKL130	(Lee *et al*, 2014)
		V69	V70	pFB262	(Bendezú *et al*, 2009)
		V71	V72	TKL130	(Lee *et al*, 2014)
		V73	V93	pTargetF	(Jiang *et al*, 2015)
		V66	V92	pTargetF	(Jiang *et al*, 2015)
pAV10	P_{PhlF}-*Cas9*-gRNA			pJF1	Gift from Eligo Bioscience
pAV14	P_{Phlf}-*sfgfp-phlF* integration in λ site	V140	V143	P_{PhlF}-*sfgfp-phlF* (synthesis)	(Stanton *et al*, 2014)
		EcoR1	Pst1	pIT5-KL (45984)	(St-Pierre *et al*, 2013)
pAV25	Integrates *pamcherry-mrdA* in the native locus	V170	V171	pSW23t	(Demarre *et al*, 2005)
		V180	V188	TKL130	(Lee *et al*, 2014)
		V185	V189	TKL130	(Lee *et al*, 2014)
pAV81	P_{127}(R20 target)-*sfgfp* integration in HK022 site.	V1	V351	AV02	(This study)
		V4	V350	AV02	(This study)
		EcoR1	Pst1	pIT5-KH (45983)	(St-Pierre *et al*, 2013)
pCas	Cas9, λ-red				(Jiang *et al*, 2015)
pE-FLP	Flippase				(St-Pierre *et al*, 2013)
pCRRNA-cos	CRISPR array cloning vector				(Cui & Bikard, 2016)

*pLC97 is available on Addgene at https://www.addgene.org/depositor-collections/bikard-crispr-repression/

Table S3. PCR primers used in this study.

Name	Sequence (5' to 3')
V1	CGCCATAAACTGCCAGGAATTGGGGATCGGCCTATGAACTGTCGACTCGAGG
V2	TTCTTCACCTTTACTCATCTAGATTTCTCCTCTTTAAAGG
V3	GGAGAAATCTAGATGAGTAAAGGTGAAGAACTGTTCACC
V4	TTAGGCGCCATGCATCTCGAGGCATGCCTGCATTATTTGTAGAGTTCATCCATGCCGTGC
V38	GGAGAAATCTAGATGGTTTCCAAGGGCGAGGAGGAT
V39	TTAGGCGCCATGCATCTCGAGGCATGCCTGCATTATTTGTACAGCTCATCCATGCC
V40	ATCCTCCTCGCCCTTGGAAACCATCTAGATTTCTCCTCTTTAAAGGAATTCC
V66	GCTGGACGTACCCGTACAGATGACAAAAAAAGCACCGACTC
V67	GAGTCGGTGCTTTTTTTGTCATCTGTACGGGTACGTCCAGC
V69	AGTAGAAAACGCAGCGGATGGTTTCCAAGGGCGAGG
V71	GCATGGATGAGCTGTACAAAACCGGTTCCGGAGGGCATG
V73	GCGGCAACGCATGATATCGGGAATTCTCTAGAGTCGACCTGCAGAAG
V75	CTGCCAGGAATTGGGGATCGGAATTCGCAACGCAATTAATGTGAG
V76	TCCTCGCCCTTGGAAACCATAGCTGTTTCCTGTGTGAAATTG
V77	ATTTCACACAGGAAACAGCTATGGTTTCCAAGGGCGAGG
V78	CATCTCGAGGCATGCCTGCATTATTTGTACAGCTCATCCATGCC
V101	TGGTGGCTGGCACAAGTGCCCTCCAGCTTTTGTTCCCTTTAGTGAGGGTTAATTGC
V102	TGACAACAAGCATTACCGCGGCTGCAGGAATTCGATATCAAGCT
V103	TGATATCGAATTCCTGCAGCCGCGGTAATGCTTGTTGTCAG
V108	AAAGGGAACAAAAGCTGGAGGGCACTTGTGCCAGCCAC
V109	AAGGTAAAAGATCTCTCCGGCTCCAGCTTTTGTTCCCTTTAGT
V110	CCATCCGTGGCGGCTCTTCGGGCTGCAGGAATTCGATATCAAGC
V111	GATATCGAATTCCTGCAGCCCGAAGAGCCGCCACGGAT
V116	AAAGGGAACAAAAGCTGGAGCCGGAGAGATCTTTTACCTTATCGC
V140	CGCCATAAACTGCCAGGAATTGGGGATCGGATCTGATTCGTTACCAATTGACATGATACG
V143	TTAGGCGCCATGCATCTCGAGGCATGCCTGCATAGTTAACGCTGTGTACCCGGACA
V170	CTCCAGCTTTTGTTCCCTTTAG
V171	GGCTGCAGGAATTCGATATCAAG
V180	TCGATAAGCTTGATATCGAATTCCTGCAGCCgccgtgccatcggggtc
V185	AACCCTCACTAAAGGGAACAAAAGCTGGAGcagtcacgataacgtttttccg
V188	CAGTTTGGCGGTCTGGGTGCCTTCATACGGACGGCCCTCGCCTTCACCTTCG
V189	GCCGTCCGTATGAAGGCACCCAGACCGCCAAACTGAAGGTGACGAAGGGTGGTC
V350	TCTGGGTGCCTTCATACGGAAGGAGAAATCTAGATGAGTAAAGGTG
V351	TCCTTCCGTATGAAGGCACCCAGACATTATAGGTATCCATGAGGTACCTG
LC100	GCAGGACGCCCGCCATAAACTGCCAGGAATTGGGGATCGGTTAAGACCCACTTTCACATTTAAG
LC283	TCCATTTTTGCCTCCTAACTAGGTCATTTGATATGCCTCC
LC284	CCTAGTTAGGAGGCAAAAATGGATAAGAAATACTCAATAGGC
LC285	AGTTTAGGTTAGGCGCCATGCATCTCGAGGCATGCCTGCAATGCCTGGAGATCCTTACTC

Table S4. Oligonucleotides pairs ligated in vectors to construct the CRISPR arrays.

Name	Primer 1	Primer 2
**First position in pCRRNA-cos**		
G20	aaacCCTTCACCTTCACCACGAACAGAGAATTTGgttttagagctatg	aacagcatagctctaaaacCAAATTCTCTGTTCGTGGTGAAGGTGAAGG
G14	aaacCCTTCACCTTGTGGTGGAACAGAGAATTTGgttttagagctatg	aacagcatagctctaaaacCAAATTCTCTGTTCCACCACAAGGTGAAGG
G11	aaacCCTTCACCTTGTGGTGCTTCAGAGAATTTGgttttagagctatg	aacagcatagctctaaaacCAAATTCTCTGAAGCACCACAAGGTGAAGG
G10	aaacCCTTCACCTTGTGGTGCTTGAGAGAATTTGgttttagagctatg	aacagcatagctctaaaacCAAATTCTCTCAAGCACCACAAGGTGAAGG
C	aaacATCGCACATCCTGGTCGCGACATTAAGAGTgttttagagctatg	aacagcatagctctaaaacACTCTTAATGTCGCGACCAGGATGTGCGAT
R20	aaacAGTTTGGCGGTCTGGGTGCCTTCATACGGAgttttagagctatg	aacagcatagctctaaaacTCCGTATGAAGGCACCCAGACCGCCAAACT
R11	aaacAGTTTGGCGGAGACCCACGCTTCATACGGAgttttagagctatg	aacagcatagctctaaaacTCCGTATGAAGCGTGGGTCTCCGCCAAACT
**Second position in pCRRNA-cos**		
R20	ctgttttgaatggtcccaaaacAGTTTGGCGGTCTGGGTGCCTTCATACGGAg	aaaacTCCGTATGAAGGCACCCAGACCGCCAAACTgttttgggaccattcaa
R18	ctgttttgaatggtcccaaaacAGTTTGGCGGAGTGGGTGCCTTCATACGGAg	aaaacTCCGTATGAAGGCACCCACTCCGCCAAACTgttttgggaccattcaa
R11	ctgttttgaatggtcccaaaacAGTTTGGCGGAGACCCACGCTTCATACGGAg	aaaacTCCGTATGAAGCGTGGGTCTCCGCCAAACTgttttgggaccattcaa
C	ctgttttgaatggtcccaaaacATCGCACATCCTGGTCGCGACATTAAGAGTg	aaaacACTCTTAATGTCGCGACCAGGATGTGCGATgttttgggaccattcaa
**Arrays of 3 guide RNAs in pCRRNA-cos**		
G20*	aaacCCTTCACCTTCACCACGAACAGAGAATTTGgttttagagctatg	aacagcatagctctaaaacCAAATTCTCTGTTCGTGGTGAAGGTGAAGG
C	ctgttttgaatggtcccaaaacATCGCACATCCTGGTCGCGACATTAAGAGTgttttaga	atagctctaaaacACTCTTAATGTCGCGACCAGGATGTGCGATgttttgggaccattcaa
R20	gctatgctgttttgaatggtcccaaaacAGTTTGGCGGTCTGGGTGCCTTCATACGGAg	aaaacTCCGTATGAAGGCACCCAGACCGCCAAACTgttttgggaccattcaaaacagc
**Arrays of 4 guide RNAs in pCRRNA-cos**		
G20*	aaacCCTTCACCTTCACCACGAACAGAGAATTTGgttttagagctatg	aacagcatagctctaaaacCAAATTCTCTGTTCGTGGTGAAGGTGAAGG
C (a)	ctgttttgaatggtcccaaaacATCGCACATCCTGGTCGCGACATTAAGAGTgttttagagcta	cagcatagctctaaaacACTCTTAATGTCGCGACCAGGATGTGCGATgttttgggaccattcaa
C (b)	tgctgttttgaatggtcccaaaacATCGCACATCCTGGTCGCGACATTAAGAGTgttttaga	atagctctaaaacACTCTTAATGTCGCGACCAGGATGTGCGATgttttgggaccattcaaaa
R20	gctatgctgttttgaatggtcccaaaacAGTTTGGCGGTCTGGGTGCCTTCATACGGAg	aaaacTCCGTATGAAGGCACCCAGACCGCCAAACTgttttgggaccattcaaaacagc
**Single-guide RNA for pAV10**		
cat	agcaTATTCTCAATAAACCCTTTA	aaacTAAAGGGTTTATTGAGAATA

*May be replaced with G11 to make pCRRNA-G11-C-C-R20.

Appendix references

Abbas A, Morrissey JP, Marquez PC, Sheehan MM, Delany IR & O'Gara F (2002) Characterization of Interactions between the Transcriptional Repressor PhlF and Its Binding Site at the phlA Promoter in Pseudomonas fluorescens F113. *J. Bacteriol.* **184:** 3008–3016

Baba T, Ara T, Hasegawa M, Takai Y, Okumura Y, Baba M, Datsenko KA, Tomita M, Wanner BL & Mori H (2006) Construction of Escherichia coli K-12 in-frame, single-gene knockout mutants: the Keio collection. *Mol. Syst. Biol.* **2:** 2006.0008

Bendezú FO, Hale CA, Bernhardt TG & de Boer PAJ (2009) RodZ (YfgA) is required for proper assembly of the MreB actin cytoskeleton and cell shape in E. coli. *EMBO J.* **28:** 193–204

Bikard D, Jiang W, Samai P, Hochschild A, Zhang F & Marraffini LA (2013) Programmable repression and activation of bacterial gene expression using an engineered CRISPR-Cas system. *Nucleic Acids Res.* **41:** 7429–7437

Cho H, Wivagg CN, Kapoor M, Barry Z, Rohs PDA, Suh H, Marto JA, Garner EC & Bernhardt TG (2016) Bacterial cell wall biogenesis is mediated by SEDS and PBP polymerase families functioning semi-autonomously. *Nat. Microbiol.* **1:** 16172

Chung CT, Niemela SL & Miller RH (1989) One-step preparation of competent Escherichia coli: transformation and storage of bacterial cells in the same solution. *Proc. Natl. Acad. Sci.* **86:** 2172–2175

Cui L & Bikard D (2016) Consequences of Cas9 cleavage in the chromosome of Escherichia coli. *Nucleic Acids Res.* **44:** 4243–4251

Demarre G, Guérout A-M, Matsumoto-Mashimo C, Rowe-Magnus DA, Marlière P & Mazel D (2005) A new family of mobilizable suicide plasmids based on broad host range R388 plasmid (IncW) and RP4 plasmid (IncPalpha) conjugative machineries and their cognate Escherichia coli host strains. *Res. Microbiol.* **156:** 245–255

Depardieu F, Didier J-P, Bernheim A, Sherlock A, Molina H, Duclos B & Bikard D (2016) A Eukaryotic-like Serine/Threonine Kinase Protects Staphylococci against Phages. *Cell Host Microbe* **20:** 471–481

Jiang Y, Chen B, Duan C, Sun B, Yang J & Yang S (2015) Multigene Editing in the Escherichia coli Genome via the CRISPR-Cas9 System. *Appl. Environ. Microbiol.* **81:** 2506–2514

Lee TK, Tropini C, Hsin J, Desmarais SM, Ursell TS, Gong E, Gitai Z, Monds RD & Huang KC (2014) A dynamically assembled cell wall synthesis machinery buffers cell growth. *Proc. Natl. Acad. Sci. U. S. A.* **111:** 4554–4559

Pósfai G, Kolisnychenko V, Bereczki Z & Blattner FR (1999) Markerless gene replacement in Escherichia coli stimulated by a double-strand break in the chromosome. *Nucleic Acids Res.* **27:** 4409–4415

Qi LS, Larson MH, Gilbert LA, Doudna JA, Weissman JS, Arkin AP & Lim WA (2013) Repurposing CRISPR as an RNA-guided platform for sequence-specific control of gene expression. *Cell* **152:** 1173–1183

Stanton BC, Nielsen AAK, Tamsir A, Clancy K, Peterson T & Voigt CA (2014) Genomic mining of prokaryotic repressors for orthogonal logic gates. *Nat. Chem. Biol.* **10:** 99–105

St-Pierre F, Cui L, Priest DG, Endy D, Dodd IB & Shearwin KE (2013) One-Step Cloning and Chromosomal Integration of DNA. *ACS Synth. Biol.* **2:** 537–541
